# Street life and pedestrian activities in smart cities: opportunities and challenges for computational urban science

**DOI:** 10.1007/s43762-021-00024-9

**Published:** 2021-11-27

**Authors:** Zhuangyuan Fan, Becky P.Y. Loo

**Affiliations:** Department of Geography, University of Hong Kong, School of Geography and Environment, Jiangxi Normal University, Nanchang, China

**Keywords:** Pedestrian Activities, GPS, Video, Wi-Fi, Bluetooth

## Abstract

Ongoing efforts among cities to reinvigorate streets have encouraged innovations in using smart data to understand pedestrian activities. Empowered by advanced algorithms and computation power, data from smartphone applications, GPS devices, video cameras, and other forms of sensors can help better understand and promote street life and pedestrian activities. Through adopting a pedestrian-oriented and place-based approach, this paper reviews the major environmental components, pedestrian behavior, and sources of smart data in advancing this field of computational urban science. Responding to the identified research gap, a case study that hybridizes different smart data to understand pedestrian jaywalking as a reflection of urban spaces that need further improvement is presented. Finally, some major research challenges and directions are also highlighted.

## Introduction

In an oil painting series crafted in early 1897, a French painter Camille Pissarro recorded the urban life of boulevard Montmartre from February to April in which “carriages, omnibuses, people, between big trees and big houses”^1^ were “surveyed” from his hotel window. The painter carefully documented different views of the boulevard in various weather conditions at varying times of the day. A hundred years after the debut of “The Boulevard Montmartre on a Winter Morning,” pervasive digital technologies and the staggering amount of data enabled researchers to repeat Pissarro’s survey at a much larger scale. In the meantime, the mounting pressure to improve pedestrian safety, enhance meaningful social interaction in public spaces, and inform public health responses further expanded interests in developing advanced computational methods to analyze the digital data, including mobile phone data, video data, and hybrid sensor data collected either passively by observations or actively through respondents’ direct participation.

In view of this deluge of urban information and efforts in deciphering the underlying urban dynamics, several papers have reviewed the use of various emerging datasets in studying pedestrian-related behaviors. For instance, Feng et al. (2021) covered a wide range of data collection methods for featuring pedestrian behaviors with a traffic engineering hierarchical structure. Shi et al. (2018) reviewed empirical data collection methods in studying crowd movement complexity based on a vehicular traffic framework. Lovreglio and Kinateder (2020) reviewed the use of augmented reality for pedestrian evacuation. Grantz et al. (2020) summarized the use of mobile phone data in informing COVID-19 responses. However, a review based on the pedestrian experience is still lacking. This paper contributes to the existing literature on pedestrian experience through a systematic review of the environment components, pedestrian behaviour, and smart data in different parts of the world. The limitations of using static environmental variables in analyzing pedestrian-crossing behaviour at road junctions only have been overcome through a pedestrian jaywalking study that integrates different sources of smart data, including bus dashcam, GSV images, and crowd-sourced platforms. Two explanatory models that incorporate traffic conditions, design, and destinations have been developed to study the spatio-temporal pattern of jaywalking. The results help to inform pedestrian-friendly design and contributes to the ultimate goal of walkable cities.

The remainder of the paper is organized as follows. Section 2 introduces the people-oriented and place-based rationale of using human behaviors and people’s appropriation of public spaces to measure street life performance and the pedestrian experience (Whyte and et al. 1980; Jacobs 2016; Gehl 1971; Loo 2021). Section 3 summarizes the existing literature. Section 4 presents a case study corresponding to the identified research gap. Finally, Section 5 concludes the findings and suggests future research directions.

## Literature background

**Fig. 1:**
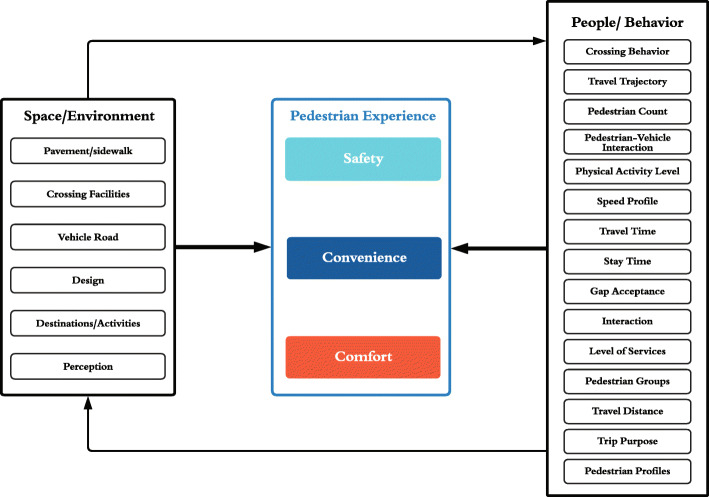
The conceptual framework

Big data and advancements in algorithms that allow us to capture many aspects of human activities are relatively new. However, concepts such as “sidewalk ballet,” livable street, walkability, and transit-oriented development can be dated back to the 1970s (Gehl 1971; Jacobs 2016). In theory, the placed-based and people-oriented thinking could be traced to planning theories advocating using human activities to measure the performance of cities (Pushkarev 1975; Whyte and et al. 1980). With the fundamental understanding of transport as an experience, pedestrians are concerned about the safety, comfort, and convenience of dwelling in streets and moving around in cities (Loo 2021). On the one hand, the experience is being determined by the interaction of people and environment directly and through the activities that people do. On the other hand, these activities are affected by the environment. For instance, the existence of a bench on the sidewalk invites people to sit and chat on the street directly. Figure 1 shows the conceptual framework of this paper.

Following Loo (2021), safety, comfort, and convenience are three basic dimensions in capturing the pedestrian experience. Pedestrian safety is predominantly measured by the number of pedestrian-vehicle crashes, street crimes, and fall hazards such as ill-maintained pavement (Abley and Turner 2011; Brown et al. 2010; Forsyth 2015; Loo and Lam 2012). The environmental components determining pedestrian safety include pavement surface, pedestrian guardrails, steps, lighting, land uses, pedestrian exposures, etc. Comfort is directly associated with the physical conditions of urban design elements such as the sidewalk width, shelter, availability of urban furniture, street lights, parks, and vegetation (Loo 2021; Forsyth 2015). Lastly, convenience involves both accessibility and mobility that facilitate pedestrian movement efficiently and smoothly to reach destinations of relevance to them. Clear signage, street network connectivity, continuity, and distance between destinations will all impact the pedestrian’s experience of convenience.

In terms of the environment, Gehl (1971) primarily classified it into buildings and space between buildings. Alternatively, one may consider the pedestrian environment to be comprised of private and public spaces. A finer division is to consider the six components of the walking environment as pavements, pedestrian cross-facilities, roads (for vehicular traffic), walkway/sidewalk design, pedestrian perceptions, and destinations/places of activities within reach (Loo 2021). Traditionally, most of these components have been measured by static indicators, such as sidewalk width and the presence/absence of street furniture. Some notable walkability assessment studies include Abley and Turner (2011); Blečić et al. (2020); Cain et al. (2014), Clifton et al. (2007), Loo (2021), and Sarkar et al. (2015). In smart cities, our knowledge regarding the effectiveness of these elements can now be measured. For instance, sidewalk width can be considered in relation to the actual pedestrian volume to detect pedestrian crowding. The ways how pedestrians actually use the street in different spatial and temporal contexts can now be captured with the availability of the Internet of Things (IoT), such as weather and pollution sensors, in cities (Loo and Tang 2019). All these geo-referenced digital data are generated 24/7 in cities and can allow researchers to experiment with the “big data” approaches and use cities as “laboratories” for urban interventions, such as the introduction of “parklets (Von Schönfeld and Bertolini 2017).”

As to pedestrian activities or behavior, recent studies in transport geography and urban planning explored data generated by individuals while they engage in daily activities like walking, shopping, making a call, taking transit, or sending a message. Some measures of pedestrian activities include pedestrian crossing behavior, travel speed, travel time, travel distance, trajectory, trip purpose, pedestrian count, physical activity level, street crime, and social interaction. In the past, pedestrian activities or behavior are predominantly captured through field observations, travel surveys, and interviews (Andrews et al. 2012; Forsyth 2015; Ramakreshnan et al. 2020; Wang and Yang 2019). Nowadays, information about these dynamic activities can be captured by location-based services (LBS) bounded with smartphone applications (APP). The pings with GPS coordinates generated by these Apps can be anonymized and aggregated to produce population-level insights on pedestrian mobility patterns (Grantz et al. 2020; Hunter et al. 2021). In the meantime, infrastructure and amenities, such as points of interest (POIs) in cities, while serving for their own purpose, also generate useful information like the number of visits or open comments that could be used to infer about human activities on streets (Hidaka and Yamamoto 2021). Videos from surveillance cameras and dashcams include a timestamp for each frame (Daamen et al. 2016). These temporal data allow researchers to analyze time-variant pedestrian activities to understand the dynamics of urban life. Similarly, an analysis of Wi-Fi signals received from individual smartphone devices have proven to be a powerful tool for detecting people travelling in groups (Huang et al. 2021; Traunmueller et al. 2018). Lastly, many researchers also develop opt-in smartphone APPs and recruit participants to record their daily mobility activities that could be associated with activity locations and individual characteristics (Zhang et al. 2021b). In smart cities, the means of collecting information about pedestrian behavior has flourished exponentially, creating huge opportunities for researchers in computational urban science to conduct innovative street life and pedestrian research.

## Advances in studying pedestrian experience through smart data

To conduct a systematic review of the advances of street life and pedestrian activity research in computational urban science, this study focuses on published peer-reviewed academic papers held within Scopus and the Web of Science. A set of keywords was used to search through the database. Following our conceptual framework (Fig. 1), space/environment, pedestrian experience, and pedestrian behavior are captured by a set of keywords^2^. Then, we combine the data source keywords, namely “video,” “GPS,” “Bluetooth,” and “Wi-Fi” with each of the keywords to create search strings and interrogate each database. Using advanced search criteria, we restricted the source type to English full-text conference papers, journal articles, and articles in press published from 1 January 2017 to May 2021. Then, the titles, abstracts, and keywords of the papers were screened based on two criteria: 1) with a description of the data collection and processing method; 2) report empirical findings on pedestrian behavior. Pure advancement in computer algorithms and tools are excluded from the selection because they do not yet help evaluate the environment-people interaction to yield insights about the pedestrian experience (see Fig. 1). Using search strings, 549 and 917 potentially relevant studies were retrieved from the Web of Science and Scopus, respectively. After identifying duplicates, abstracts were checked for conformity with the search criteria. After the evaluation, 42 papers, as shown in Table 1 remain on the list.

**Table 1:** New advances in studying pedestrian experience through smart data

Pedestrian Experience	No.	Paper	Environmental Component	Pedestrian Behavior	Smart Data
Safety	1	(Sheykhfard and Haghighi 2020)	Crossing facilities, Vehicular road	Gap acceptance, Crossing behavior, Pedestrian groups, Pedestrian-vehicle interaction	Camera - fixed & dashcam
	2	(Avinash et al. 2019)	Vehicular road, Crossing facilities	Crossing behavior, Speed profile	Camera - fixed - by researcher
	3	(Mukherjee and Mitra 2019)	Crossing facilities, Vehicular road, Perception	Crossing behavior, Pedestrian-vehicle interaction, Pedestrian count	Camera - fixed - by researcher
	4	(Beitel et al. 2018)	Design, Vehicular road	Travel trajectory, Pedestrian-bike interaction, Pedestrian count	Camera - fixed - by researcher
	5	(Kadali and Vedagiri 2020)	Crossing facilities	Speed profile, Pedestrian profiles	Camera - fixed - by researcher
	6	(Gitelman et al. 2019)	Crossing facilities	Crossing behavior	Camera - fixed - by researcher
	7	(Zhao et al. 2019)	Crossing facilities, Vehicular road	Gap Acceptance, Crossing behavior	Camera - fixed - by researcher
	8	(Liang et al. 2021)	Design, Vehicle Road	Pedestrian-bike interaction	Camera - fixed - by researcher
	9	(Zhang et al. 2020)	Vehicular Road	Crossing behavior	Camera - fixed - by researcher
	10	(Fu et al. 2019)	Crossing facilities, Vehicular road	Pedestrian-vehicle interaction	Camera - fixed - by researcher
	11	(Marisamynathan and Vedagiri 2017)	Crossing facilities, Perception	LOS, Speed profile	Camera - fixed - by researcher
	12	(Gorrini et al. 2018)	Vehicular Road, Design, Crossing facilities, Pavement, Perception	Pedestrian-vehicle interaction, LOS, speed profile, Crossing behavior, Gap acceptance	Camera - fixed - by researcher
	13	(Saleh et al. 2020)	Crossing facilities	Crossing behavior	Camera - fixed - by researcher
	14	(Choi et al. 2019)	Crossing facilities, Perception	Crossing behavior	Camera - fixed - by researcher
	15	(Arhin et al. 2021)	Crossing facilities	Crossing behavior	Camera - fixed - by researcher
	16	(Stipancic et al. 2021)	Crossing facilities	Travel trajectory, Crossing behavior, Pedestrian-vehicle interaction	Camera - fixed - by researcher
	17	(Noh et al. 2020)	Crossing facilities, Destination/Activities	Travel trajectory	Camera - fixed - CCTV
	18	(Zuo et al. 2021)	Destination/Activities	Pedestrian count, Pedestrian groups, Interaction	Camera - fixed - CCTV
	19	(Zhu et al. 2021)	Vehicular road	Crossing behavior	Camera -fixed - CCTV
	20	(Zhang et al. 2021a)	Destination/Activities, Perception	Street crime, Pedestrian visits	GPS - passive - multi-app
Comfort	21	(Cheng et al. 2019)	Design	Pedestrian groups	Camera - fixed - CCTV
	22	(Kaparias and Wang 2020)	Vehicular road, Design, Perception	LOS	Camera - fixed - by researcher
	23	(Zandieh et al. 2017)	Destination/Activities, Design	Physical activity level	GPS - apps
	24	(Hidaka and Yamamoto 2021)	Destination/Activities, Design	Stay time, Travel time	GPS - apps & Bluetooth
	25	(Athey et al. 2020)	Destination/Activities	Interaction, Stay time	GPS - passive - multi-app
	26	(Li et al. 2019)	Destination/Activities, Design	Travel trajectory	GPS - wearable
	27	(James et al. 2017)	Design	Physical activity level	GPS - wearable
	28	(Jansen et al. 2017)	Design	Speed profile, Travel time	GPS - wearable
Convenience	29	(Yoshimura et al. 2017)	Destination/Activities	Travel trajectory, Travel time	Bluetooth sensor
	30	(Hidayati et al. 2020)	Destination/Activities, Design	Interaction	Camera - movable - by researcher
	31	(Prescott et al. 2021)	Pavement, Design	Travel trajectory	Camera - movable - fixed at participants’ head
	32	(Yamamoto et al. 2018)	Destination/Activities, Design	Travel distance, Travel trajectory	GPS - apps
	33	(Remmers et al. 2019)	Destination/Activities, Pavement	Physical activity level	GPS - wearable
	34	(Filomena and Verstegen 2021)	Destination/Activities, Design	Travel trajectory, Pedestrian count	GPS from 3d party
	35	(Fukuyama 2020)	Destination/Activities, Design, Pavement, Vehicular road	Travel trajectory, Stay time, Travel time	GPS probe-person survey
	36	(Traunmueller et al. 2018)	Destination/Activities, Design	Travel trajectory	Wi-Fi probes
Convenience & Comfort	37	(Salazar Miranda et al. 2021)	Destination/Activities, Design, Pavement	Travel trajectory, Travel distance, Trip purpose	GPS - passive - multi-app
	38	(Hunter et al. 2021)	Destination/Activities, Design	Trip purpose, Travel distance	GPS - passive - multi-app
	39	(Mizzi et al. 2018)	Destination/Activities, Design	Pedestrian count, Travel trajectory	GPS - passive - multi-app
	40	(Williams et al. 2018)	Destination/Activities, Design, Pavement	Physical activity level	GPS - wearable
	41	(Lee 2020)	Destination/Activities, Design, Pavement	Physical activity level	GPS - wearable
Comfort & Safety	42	(Hahm et al. 2019)	Vehicular road, Design	Travel time, Stay time	GPS - apps

In Table 1, we group the literature based on the pedestrian experience of safety, comfort, and convenience. Next, we retrieve the key environmental components and pedestrian behaviors measured in each study. Specifically, the environmental components are categorized into six major types: design, destination and activities, crossing facilities, vehicular road, pavement, and pedestrian perception (Loo 2021). The types of pedestrian behavior measures are identified. Lastly, we highlight the type of smart data used. So far, what observations can we make about the recent advances in street life and pedestrian activities in smart cities? Here, we used a number of alluvial diagrams to answer this question from the perspectives of environmental components, pedestrian activities, and sources of smart data.

### Measure of pedestrian activities

**Fig. 2:**
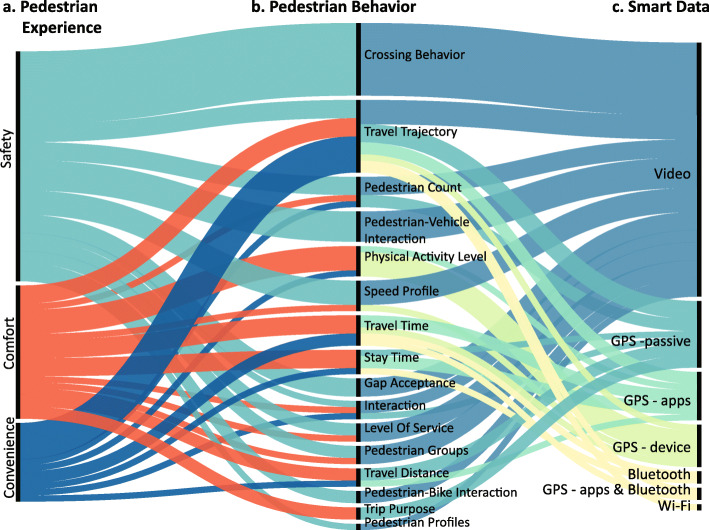
Distribution of smart data, pedestrian behavior measures and pedestrian experience in the reviewed paper. **a** Pedestrian experience factors; **b** Pedestrian behaviors measured in each paper; **c** Smart data used in each paper

In Fig. 2, we first summarize how the pedestrian experience (a) has been captured by different behavioral measures (b) and the type of smart data used (c). Under the pedestrian behavioral measures, pedestrian crossing behaviors and travel trajectories are the most the frequent behavioral measures. The crossing behavior, predominantly measured from video data, is widely studied to measure pedestrian safety. For example, Mukherjee and Mitra (2019) captured the state of crossing - using an electronic device or carrying loads - of each observed pedestrian at 24 intersections in Kolkata city, India. Choi et al. (2019) measured older pedestrians’ start-up delay, the head-turns frequency at 30 pedestrian crossings in Seoul, South Korea. Other crossing-relevant behaviors such as waiting time before crossing and gap acceptance time are also frequently measured in these studies (Sheykhfard and Haghighi 2020; Zhang et al. 2020; Zhao et al. 2019).

Travel trajectories have been used to evaluate all three aspects of the pedestrian experience. For example, Noh et al. (2020) derived pedestrian trajectories at un-signalized crosswalks through videos from road security cameras and classified crosswalk locations by their safety levels. Traunmueller et al. (2018) gathered devices scanned at each Wi-Fi access point (AP), inferred the pedestrian travel trajectories, and identified the most frequently used street segments in Manhattan. Salazar Miranda et al. (2021) and Hunter et al. (2021) used passive GPS data to derive regional aggregated travel trajectories and discussed pedestrian’s route choice in relationship to the level of convenience and comfort of the pedestrian network. In addition, some researchers recruited participants to wear GPS devices or install applications on their smartphones to collect travel trajectories for specific groups of interests (Fukuyama 2020; Hahm et al. 2019; James et al. 2017; Jansen et al. 2017; Li et al. 2019; Williams et al. 2018; Yamamoto et al. 2018; Zandieh et al. 2017). Pedestrian activity level is another frequently measured variable. These studies mostly relied on distributing GPS devices to participants and then gathering smart data about their daily activity pattern and level of activities (Jansen et al. 2017; Remmers et al. 2019; Lee 2020).

Also, pedestrian grouping and social interaction are two crucial variables that determine the quality of street life. Hidayati et al. (2020) record social interaction along streets within an informal settlement in Jakarta, Indonesia. Sheykhfard and Haghighi (2020) studied pedestrian group size and its influence on pedestrian crossing behaviors. Related to social interaction, Athey et al. (2020) used passive-GPS data to calculate census tract level visitors’ racial segregation. However, these studies do not directly observe the interaction. Instead, they assume that people who share the same spatial units within a specific time range have the potential to interact with each other. In addition, the recent pandemic of COVID-19 also leads to a surge of studies using Bluetooth, Wi-Fi traces, and smartphone applications to inferring individual-level contact and social distancing (Berke et al. 2003; Faggian et al. 2020). To conclude, these are limited studies focusing on encouraging positive and meaningful street-level social interaction using the smart data, especially considering the recent development in group detection algorithms (Cheng et al. 2019; Zaki and Sayed 2018).

In contrast with the measures mentioned above, pedestrian profiles, such as gender, age, and income level, are not directly accessible from most smart data. However, some studies using video data hired trained observers to annotate each pedestrian per frame (Arhin et al. 2021; Kaparias and Wang 2020; Liang et al. 2021; Saleh et al. 2020; Zhu and Sze 2021). Fukuyama (2020) also asked participants to fill in their profiles when they agreed to participate in the study. For studies using passive GPS data in the U. S., given the country is generally segregated in terms of income and race, researchers would associate the inferred home census tract of each device and use the census survey income level and race distribution to derive the pedestrian profile (Hunter et al. 2021; Aleta et al. 2020).

### Spatial-temporal resolution for capturing pedestrian behavior

**Fig. 3:**
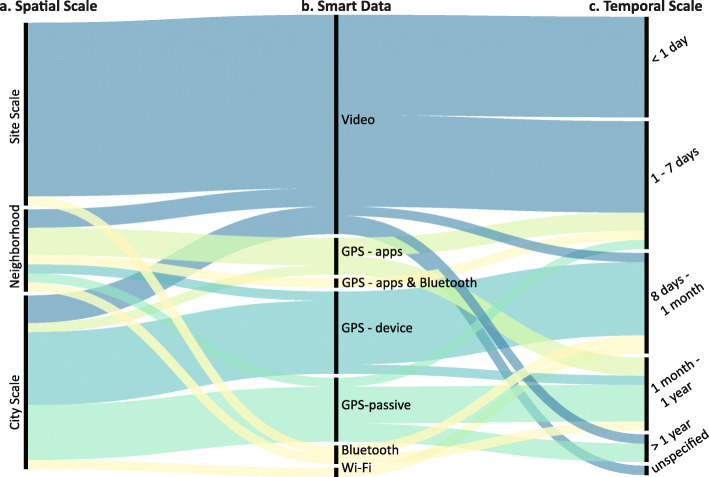
The spatial-temporal scales of reviewed studies using different data sources. **a** Spatial scales; **b** Smart data used in each study; **c** Temporal Scales. Colors correspond to each type of main data source

One of the key advantages of emerging big urban data in smart cities is the fine-grained spatial-temporal resolution and large sample size. Among all reviewed studies, pedestrian activities have been captured at the macro level of cities and regions, at the meso level of neighborhoods and districts, and the micro-level of streets segments and intersections. The time interval can range from a couple of hours to several years. In Fig. 3, research papers listed in Table 1 are analyzed by (a) the spatial scale, (b) data sources, (c) and the temporal scale.

Several observations can be made. First, video data are predominantly collected at the site level, and the observation time ranges from hours to days. Studies using such data are capable of capturing individual characteristics and fine-scale behavior such as head turns during crossing (Choi et al. 2019), pedestrian-bike interactions (Beitel et al. 2018; Liang et al. 2021), and load carrying (Mukherjee and Mitra 2019). Second, recent developments in deep learning algorithms have gradually enlarged the scope of studies using video data. The observation time of studies using video editing software or trained observers ranged from two hours to three days. In contrast, Noh et al. (2020) used the Mask R-CNN (He et al. 2018) algorithm to detect pedestrian crossing behaviors with 400 days of closed-circuit television videos. Similarly, Zuo et al. (2021) applied Yolo (Redmon and Farhadi 2018) to videos collected from 731 locations in New York City to identify pairs of pedestrians that violate the social distancing regulation.

GPS data collected from researcher-designed APPs or distributed GSP devices could cover a much larger spatial-temporal range. However, the challenges of recruiting participants often result in small sample sizes. Hence, they predominantly focused on specific pedestrian groups such as teenagers (Lee 2020; Williams et al. 2018) or older people (Li et al. 2019; Zandieh et al. 2017). In addition, Bluetooth and Wi-Fi-scanner have been installed at specific sites such as festival sites, historic neighborhoods, and dedicated neighborhoods (Traunmueller et al. 2018; Yoshimura et al. 2017; Zhang et al. 2020; Yoshimura et al. 2017) to capture the dynamics of street life. However, considering the high privacy concern, they are only applicable at limited locations and for a limited period of time.

Passive-GPS data collected from mobile phones has the advantage of yielding comprehensive spatial-temporal coverage as well as a large sample size. Hunter et al. (2021), and Athey et al. (2020) used mobility data collected from millions of unique devices from all over the U.S. These studies provided general knowledge on aggregated population-level pedestrian behavior. However, it is still worth noticing the potential sample bias as smartphone users are generally at a younger age. Demographic groups such as seniors, children, and low-income populations are usually under-represented.

### Evaluation of the pedestrian environment

**Fig. 4:**
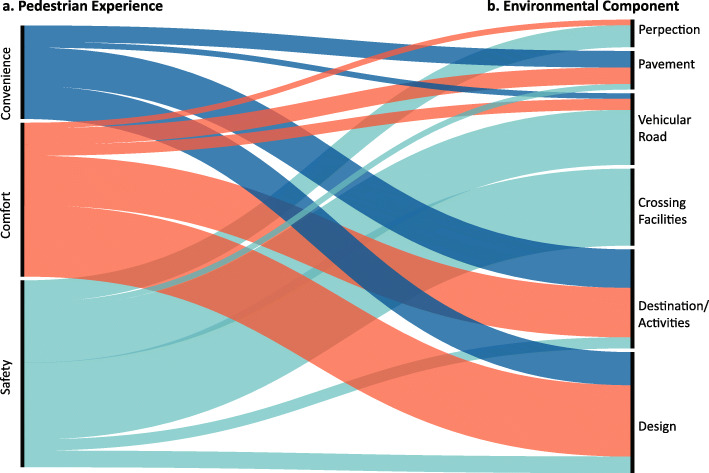
Distribution of measured environmental components and their associated pedestrian experience variables in the reviewed studies

Lastly, we turn to the urban environment components that are shown to have an impact on the pedestrian experience. Figure 4 shows (a) dimensions of pedestrian experience with (b) the environmental factors, such as pavement, vehicular road, crossing facilities, destinations/activities, and design according to six components of the walking environment described under our conceptual framework.

Regarding pedestrian safety, variables related to vehicle roads and crossing facilities are most frequently studied. Notably, traffic volume, traffic speed, vehicular gap size, and perceived distance of an approaching vehicle are shown to be key factors influencing pedestrian’s crossing decision, crossing speed, trajectory, gap acceptance time, red-light running, interaction with bikes and cars, etc. (Fu et al. 2019; Kadali and Vedagiri 2020; Zhang et al. 2020). The crossing facilities variables include crosswalk marking, pedestrian signage, the existence of refugee island, and crosswalk length, etc. (Noh et al. 2020; Saleh et al. 2020; Zhang et al. 2020). In addition, pavement and design features such as sidewalk continuity, accessible ramp, and the design of shared space for pedestrians and cyclists are also discussed (Kaparias and Wang 2020; Gorrini et al. 2018; Liang et al. 2021).

Design, destination, and activity components are mostly discussed in relation to pedestrian comfort. Destinations such as parks, plazas, shops, restaurants, transit stations, and schools are shown to influence pedestrian density, trajectories, route choice, stay time, walking speed, physical activity level, and level of social mixing. Design variables include available urban furniture, sidewalk width, sidewalk sinuosity, and greenness (James et al. 2017; Salazar Miranda et al. 2021). Moreover, several studies also discussed pedestrian activity and special events. For example, Yoshimura et al. (2017) studied the pedestrian path patterns concerning the sales events in Barcelona, Spain. Mizzi et al. (2018) discussed the different mobility patterns between residents and foreigners during tourist events in Venice. Hunter et al. (2021) showed increased recreational walking during the COVID-19 in ten metropolitan areas in the U.S.

Pedestrian convenience, directly associated with mobility and accessibility, was found to be closely related to wayfinding, accessibility, network connectivity, and distance from major destinations. For example, Filomena and Verstegen (2021) modeled the effects of landmarks on pedestrian volumes and pedestrian route choice. Hidayati et al. (2020) measured realized accessibility of an informal settlement in Jakarta and shows the lack of a walkable path to access education facilities. Distance between schools and homes is associated with children’s physical activity level (Lee 2020; Remmers et al. 2019).

It is worth mentioning that among all environmental components, pedestrian perception is a crucial variable frequently discussed in literature yet does not show consistent ways of measurement in the studies reviewed. Kaparias and Wang (2020) used Pedestrian Level of Service (PLOS) to indicate pedestrian perception scores for intersections, street segments, and links. Choi et al. (2019) conducted surveys to collect pedestrian’s perception of distance from the crosswalk to an approaching vehicle. Gorrini et al. (2018) interviewed a group of the aged population regarding the perceived walkability of their neighborhood. Besides, it is worth noting that the advancement in deep learning algorithms and computer vision methods allow researchers to predict human perception based on street view images (Salesses et al. 2013; Ordonez and Berg 2014; Helbich et al. 2019; Dubey et al. 2016). Zhang et al. (2021a) used the perception of safety predicted from Google Street Views (GSV) to explore street crimes events in Houston, U.S. Given the potential sample bias within the original training dataset, these methods are not always transferable to other geographic contexts.

To this end, by reviewing the selected paper from the measure of pedestrian activities, spatial-temporal resolution, and the environmental components, we have three overall observations. First, a great portion of studies have concentrated around pedestrian safety studies, indicating a common consensus that safety is still one of the main concerns in planning practice and research. Secondly, similar research questions tend to cluster around a similar spatial and temporal resolution, implying a necessity to further explore diverse sources of dataset with various resolutions and spatial coverage to compensate our current understanding of pedestrian experience. Lastly, the built environment features are mostly measured by their function and structure, with limited discussion on the visual quality or perception, which is a key components determining the pedestrian experience.

## Case study: safety, convenience, and comfort on pedestrian crossing behavior

In response to these observations, we pick one of the most frequently studied pedestrian measures, pedestrian crossing, and conducted a pilot project in understanding pedestrian jaywalking activities as an indicator to improve urban planning and design. As the reviewed studies have primarily focused on pedestrian crossing at road intersections, here we leverage a new data source - dashcam on buses, to capture pedestrian crossing behavior dispersed over different spatial contexts. In addition, by including derived visual features from GSV, we also hinted on the potential visual influence of urban environment on pedestrians’ likelihood to cross at different locations. Incorporating the concept of pedestrian-centered place design, a hot spot for pedestrian jaywalking suggests a fundamental need to change a vehicle-oriented design that jeopardizes pedestrian safety rather than to punish the unsafe behavior of pedestrians by enforcement only (Loo 2021). Here we train a Mask R-CNN model to detect jaywalking behaviors, highlight the unexpected jaywalking locations, and conclude with the environmental factors that are correlated with pedestrians’ jaywalking behaviors.

### Analysis framework

**Fig. 5:**
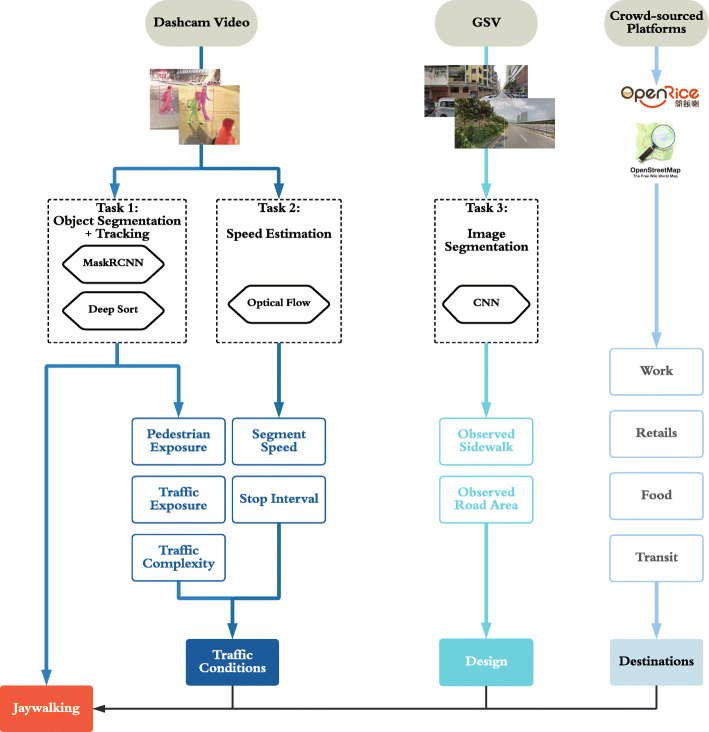
Pedestrian Jaywalking Analysis Framework

With reference to Fig. 5, we first measure three main types of variables that are found to be contributing to pedestrian jaywalking in the previous literature: traffic conditions, design and destinations. The traffic condition variables include the local pedestrian volume, traffic volume, transportation mode complexity, traffic flow speed. The design aspects measure the observed size of sidewalk and road. Lastly, the destinations include retails, transit stations, restaurants and work destinations.

### Data and method

#### Study sites and data coverage at different time periods

**Fig. 6:**
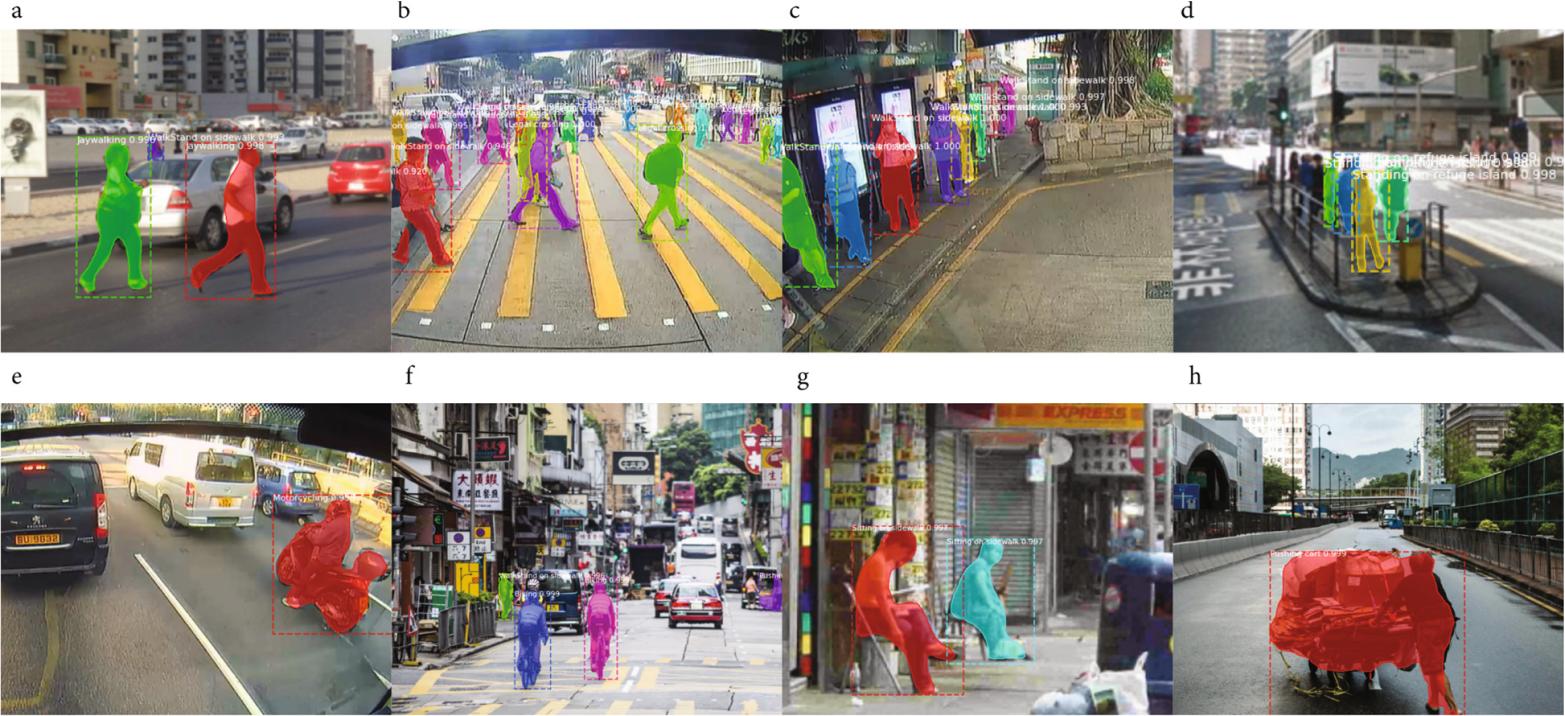
Spatial Distribution of Jaywalking Events and Detection Example. **a**, **b**, **c** Jaywalking detected from videos collected between 6 - 9 am. **d**, **e**, **f** Jaywalking detected from videos collected between 12 - 3 pm

A total of 208,171 m road segments is covered by a total of 24-hour data distributed in the morning, noon, and night time from eight major bus routes in Hong Kong. All video data are first processed with an anonymizer algorithm^3^ to blur individual faces for the sake of privacy protection. All derived parameters are aggregated at the Basic Spatial Unit (BSU) level. A BSU is a road segment of maximum 100-meter length that has been used for crash hot zone identification (Loo 2009). Night videos are not included due to the inadequate lighting. The grey line profiles in Fig. 6 show the street segments covered by the dashcam during the morning and at noon.

#### Jaywalking detection from dashcam videos

**Fig. 7:**
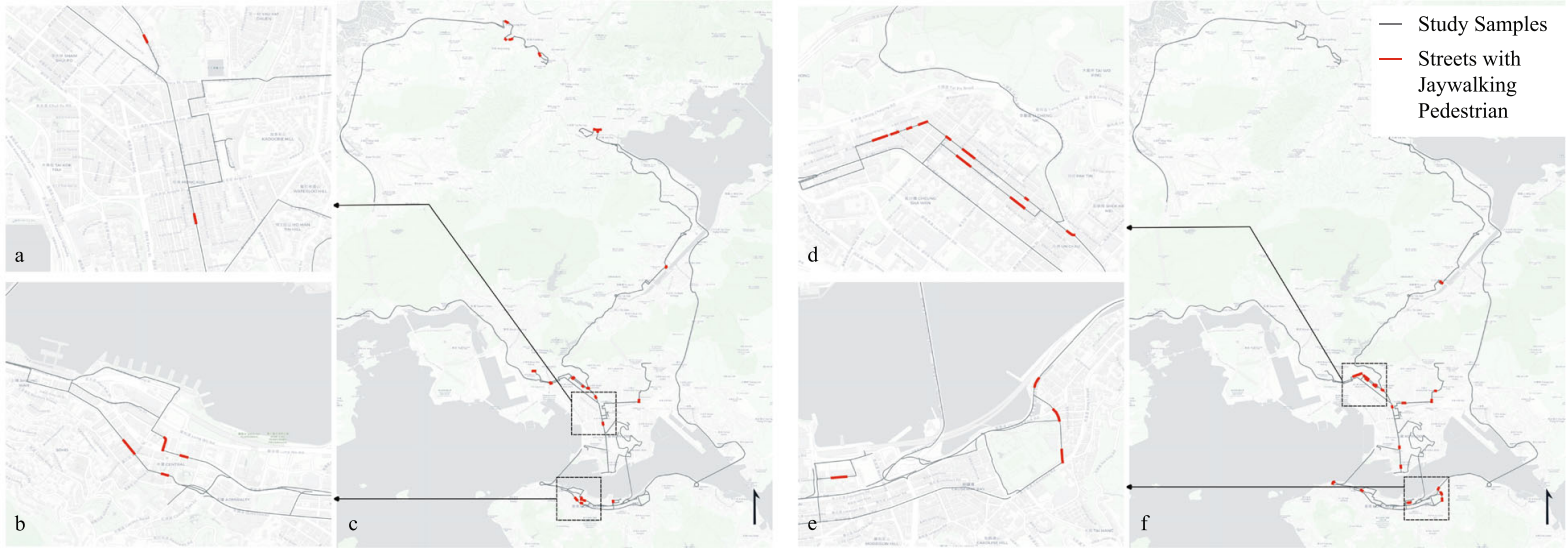
Mask R-CNN results on the validation dataset, mask mAP at 0.5 IOU (58.6). The training and validation dataset contain both image frames obtained directly from the dashcam video and images from the Internet. Mask R-CNN coco dataset mask mAP using ResNet-101-FPN backbone is 58.0, and with ResNeXt-101-FPN backbone is 60 (He et al. 2018)
- Legal crossing: pedestrians walking on the zebra crosswalk;
- Stand or walk along the sidewalk: pedestrians on the sidewalk;
- Standing on Refugee Island: pedestrian walking or standing on a refugee island;
- Motorcycling: person riding a motorcycle;
- Biking: person riding a bike;
- Pushing Cart: person pushing a cart;
- Sitting: sitting along sidewalk.

We train a Mask R-CNN model using pre-trained coco weights^4^ with 200 manually labeled images that contains 8 classes (Fig. 7):

- Jaywalking: pedestrians walking on the vehicle road;

#### Environmental factors

Previous literature has identified that pedestrian jaywalking behavior is associated with factors including the destination, traffic condition, and built environment features. Here we obtained the variables to measure these features as indicated in Table 2.

**Table 2:** Summary Statistics

	6 - 9 am				12 - 3 pm			
	mean	sd	min	max	mean	sd	min	max
Jaywalking Dummy	0.381	0.486	0.000	1.000	0.326	0.469	0.000	1.000
Jaywalking (per Frame)	0.017	0.046	0.000	0.745	0.014	0.036	0.000	0.301
Segment Length	84.113	23.144	13.537	100.000	86.996	21.067	16.587	100.000
Traffic Condition								
*#* Traffic Mode	3.176	0.832	0.000	5.000	2.986	0.878	0.000	5.000
car	1.910	1.579	0.000	8.852	1.749	1.530	0.000	8.689
Average Speed	5.277	3.109	0.131	14.545	6.570	4.806	0.118	20.985
Gap Time (min)	0.291	0.471	0.000	3.633	0.258	0.446	0.000	3.167
Ped. Vol.(h)	456.604	474.048	18.188	3860.058	485.210	574.420	14.813	5595.291
Design								
Sidewalk Index	0.050	0.033	0.000	0.170	0.046	0.033	0.000	0.170
Road Index	0.298	0.050	0.146	0.444	0.295	0.054	0.125	0.444
Destinations								
*#* Retails	0.624	1.891	0.000	15.000	0.563	1.773	0.000	15.000
*#* Food	2.740	9.183	0.000	87.000	2.544	8.154	0.000	81.000
*#* Transit	4.383	7.226	0.000	40.000	4.644	7.706	0.000	40.000
*#* Finance Inst.	0.323	0.943	0.000	7.000	0.335	0.955	0.000	7.000
Observations	535				579			

Only BSUs longer than 10 meter, with at least 20 frames sampled are included. Segments do not have any observed pedestrians are dropped

The traffic conditions are measured directly using the same dashcam video. Number of traffic modes counts the unique number of moving objects along the streets: bicycles, cars, trucks, buses, motorcycles. Average speed indicates the average moving speed of buses along each street segment. It is derived by using the total length of each segment divided by the time it takes for a bus to move through, excluding the bus stopping time. Gap time refers to the average time each bus stops during its trip through a street segment. Lastly, pedestrian volume measures the hourly total pedestrian visit in one street segment. It is derived using the Mask R-CNN model in combination with Deepsort (Wojke and Bewley 2018).

Then, the design characteristics, including observable sidewalk and road surfaces are extracted using GSV images with an image segmentation algorithm that assigns each pixel in an image to a given category (Zhu et al. 2020). These two factors are considered in the model given that both road width and sidewalk spaces are found to be correlated with pedestrian waiting time and crossing behavior in the reviewed studies. The GSV images were collected via their Application Protocol Interface (API). To collect the images, we first sampled points at 50m intervals along the OSM street centerlines within study areas. For each sampled point, we queried the google API, which returns four images of size 400 x 400 pixels. Each four-image set would constitute a street view panorama captured at a single point in time. The image segmentation model used in this study was trained using the ADE20K dataset, which has a total of 150 categories available.^5^ All the metrics constructed measure the share of pixels of each type among the total pixels in each image. Then, for each street segment, we aggregated the average observed metrics from all sampled images.

Finally, the destination data are from OpenStreetMap 6 and OpenRice 7. Each destination POI was firstly linked with a street segment by identifying their closest street segment. Then we aggregate the number of destinations by each street segment.

### Method

Considering jaywalking events are usually rare in Hong Kong, we specify two measures to estimate the level of jaywalking. The first measure is a dummy variable to define if a street segment has at least one jaywalking pedestrian during the entire observation period. The second specification measures the average number of jaywalking people observed per frame^8^ per BSU. Only BSUs that are covered by more than 20 frames of video data are included in the study. Figure 6 plots the streets with observed jaywalking people either between 6 - 9 am or 12 - 3 pm in the afternoon. For the two measures, we developed two separate models. For the dummy measure of jaywalking, we used a logistic regression model (eq 1) to understand the connection between the likelihood of pedestrian jaywalking and environmental factors. For the continuous measure of jaywalking pedestrian, we used a linear regression model (eq 2) to estimate the relationship between the intensity of jaywalking and environmental factors. Both models controlled for street type fixed-effect, adjacent population, and segment length.

1 $$ \begin{aligned} Logit_{t}(Y) &= \theta_{1} Traffic Condition_{t} + \theta_{2} Design \\&\quad+\theta_{3} Destination + \alpha +\epsilon \end{aligned}  $$

2 $$ \begin{aligned} Jaywalking_{t}(Y) &= \beta_{1} Traffic Condition_{t} + \beta_{2} Design \\&\quad+\beta_{3} Destination + \alpha +\epsilon \end{aligned}  $$

In equation 1, *Y*_*t*_ is the dummy indicating if a BSU has seen at least one jaywalking event through the video sample series during time *t*. *α* is the combined estimation of street type fixed-effect, adjacent residential population and street segment length. In equation 2, *Jaywalking*_*t*_(*Y*) is the number of jaywalking detected per frame per BSU during the time period *t*. Both models measure the effect of parameters from the lens of traffic conditions, design, and destinations.

### Results

Tables 3 and 4 summarize the model results. Among all traffic conditions factors, Table 3 columns 1 and 5 show that BSUs with a high pedestrian volume, slow road segment average speed, and longer traffic gap time has a higher likelihood of observing jaywalking pedestrians. These effects are higher in the morning than at noon, potentially since pedestrians in the morning are more time-sensitive. Similarly, columns 1 and 5 in Table 4 indicate a similar finding, except that the effect of gap time diminishes. It implies that a longer gap time of traffic increases the likelihood of jaywalking events but not necessarily increases the number of jaywalking pedestrians.

**Table 3:** Main results: Jaywalking Behaviors (Dummy) and Environmental Factors

	Jaywalking Dummy							
	6 - 10 am				12 - 3 pm			
	(1)	(2)	(3)	(4)	(5)	(6)	(7)	(8)
Traffic Condition								
*#* Traffic Mode	0.139			0.125	0.213			0.207
	(0.159)			(0.161)	(0.131)			(0.134)
Traffic Volume	-0.112			-0.065	0.029			0.104
	(0.236)			(0.241)	(0.219)			(0.237)
Average Speed	-0.110^*^			-0.118^*^	-0.116^**^			-0.114^**^
	(0.063)			(0.063)	(0.048)			(0.049)
Gap Time (min)	1.223^***^			1.169^***^	0.656^**^			0.629^*^
	(0.394)			(0.395)	(0.316)			(0.322)
Log(Pedestrian Vol.)	0.933^***^			0.814^***^	0.685^***^			0.597^***^
	(0.142)			(0.148)	(0.123)			(0.139)
Design								
Log(Sidewalk (GSV))		14.675^***^		3.179		10.798^**^		-0.191
		(4.355)		(5.324)		(4.122)		(5.122)
Log(Road (GSV))		0.911		-2.893		-5.512^*^		-2.014
		(3.581)		(4.234)		(3.151)		(3.583)
Destination								
Log(*#* Retails)			0.540^**^	0.325			0.161	0.262
			(0.255)	(0.265)			(0.261)	(0.264)
Log(*#* Food)			0.170	-0.050			0.527^***^	0.222
			(0.134)	(0.140)			(0.141)	(0.140)
Log(*#* Transit)			0.217^**^	-0.014			0.056	-0.159
			(0.092)	(0.106)			(0.090)	(0.106)
Log(*#* Finance Inst.)			0.746^**^	0.316			0.362	0.057
			(0.315)	(0.295)			(0.279)	(0.299)
Observations	505	505	505	505	550	550	550	550
R-squared	0.244	0.051	0.098	0.255	0.210	0.089	0.122	0.225
BSU Length Controlled	Yes	Yes	Yes	Yes	Yes	Yes	Yes	Yes
Street Type Fixed-Effect	Yes	Yes	Yes	Yes	Yes	Yes	Yes	Yes

Logistic regression at BSU level. Standard errors in parentheses. *#* refers to count. ^***^ denotes a coefficient significant at the 0.5*%* level, ^**^ at the 5*%* level, and ^*^ at the 10*%* level

**Table 4:** Main results: Jaywalking Behaviors (per Frame) and Environmental Factors

	Jaywalking per Frame							
	6 - 10 am				12 - 3 pm			
	(1)	(2)	(3)	(4)	(5)	(6)	(7)	(8)
Traffic Condition								
*#* Traffic Mode	0.001			0.000	0.000			0.000
	(0.002)			(0.002)	(0.002)			(0.002)
Traffic Volume	0.001			0.000	0.001			0.002
	(0.003)			(0.003)	(0.003)			(0.003)
Average Speed	0.000			0.000	-0.001^***^			-0.001^***^
	(0.001)			(0.001)	(0.000)			(0.000)
Gap Time (min)	0.007			0.007	-0.002			-0.003
	(0.007)			(0.007)	(0.004)			(0.004)
Log(Pedestrian Vol.)	0.012^***^			0.012^***^	0.006^***^			0.005^***^
	(0.002)			(0.003)	(0.001)			(0.002)
Design								
Log(Sidewalk (GSV))		0.159^**^		-0.005		0.013		-0.084
		(0.066)		(0.059)		(0.063)		(0.070)
Log(Road (GSV))		0.092		0.072		-0.153^***^		-0.099^**^
		(0.078)		(0.065)		(0.050)		(0.048)
Destination								
Log(*#* Retails)			-0.003	-0.004			-0.000	0.001
			(0.004)	(0.004)			(0.005)	(0.005)
Log(*#* Food)			0.003	0.001			0.010^***^	0.007^**^
			(0.003)	(0.003)			(0.003)	(0.003)
Log(*#* Transit)			0.003^*^	-0.000			-0.002	-0.003^*^
			(0.001)	(0.001)			(0.001)	(0.002)
Log(*#* Finance Inst.)			0.010	0.004			-0.002	-0.004
			(0.006)	(0.006)			(0.005)	(0.005)
Observations	535	535	535	535	579	579	579	579
R-squared	0.104	0.029	0.043	0.109	0.088	0.065	0.093	0.128
BSU Length Controlled	Yes	Yes	Yes	Yes	Yes	Yes	Yes	Yes
Street Type Fixed-Effect	Yes	Yes	Yes	Yes	Yes	Yes	Yes	Yes

OLS at BSU level. Standard errors in parentheses. *#* refers to count. ^***^ denotes a coefficient significant at the 0.5*%* level, ^**^ at the 5*%* level, and ^*^ at the 10*%* level

Columns 2 and 6 in both tables show the effects of design parameters on jaywalking. Consistently, BSUs with larger observable sidewalks are more likely to see a higher level of jaywalking pedestrians in the morning. This is likely due to the fact that streets with observable sidewalks tend to have many destinations on both sides of the streets. Again, this effect is more substantial in the morning. On the contrary, BSUs with more vehicular traffic are less likely to see pedestrians jaywalking at noon.

Regarding the destination parameters, columns 3 and 7 in Table 3 show that during 6 - 10 am, streets with more retails, transit stops, and financial institutions have a higher log-ratio to observe people jaywalking. At the same time, restaurants and other food-related places are more likely to observe jaywalking behaviors at noon. Columns 3 and 7 in Table 4 indicate that BSUs with more food-related services at noon are correlated with a higher level of jaywalking behaviors. BSUs with a higher number of transit stops see more intense jaywalking behaviors in the morning.

Lastly, comparing columns 4 and 8 in both tables, we find that when including all parameters together, the pedestrian volume still positively correlates with the log odds ratio of pedestrian jaywalking and the number of jaywalking behaviors. Similarly, the speed of traffic has a negative correlation with pedestrian jaywalking. The effects of design factors and destination factors diminish mainly due to the fact that streets with observable sidewalks and more destinations also have higher pedestrian volume and slower traffic speed. However, food-related services serve as a useful indicator of a higher number of jaywalking behaviors at noon. One percent increase in food services is correlated with a 0.3 percent higher number of jaywalking pedestrians per frame. On the contrary, larger vehicle-road is associated with a lower number of jaywalking behavior at noon.

### Jaywalking, safety and pedestrian-friendly design

**Fig. 8:**
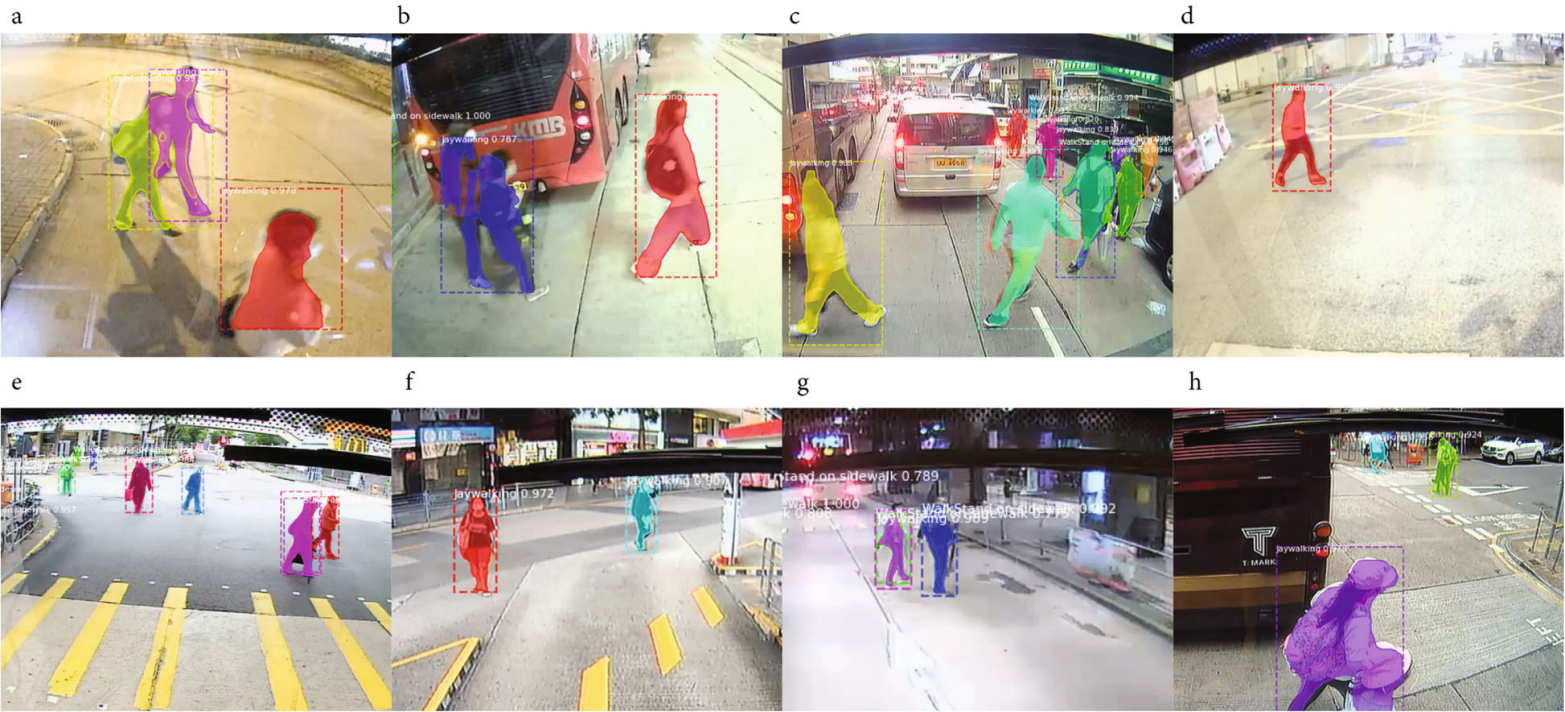
Detected jaywalking behaviors at different locations in the city
- Pedestrian jaywalk at places that they don’t agree with the urban design intention (Fig. 8e-g)
- Pedestrians are crossing where the road crossing facilities are different from traditional crossing marks (Fig. 8h).

Figure 8 shows different scenarios of jaywalking detected in all video samples. We observe three main types of detected jaywalking:

- Pedestrian jaywalk right in front of the bus when the bus is stopped at bus station, intersection, or just during congestion (Fig. 8a-d)

The first type of jaywalking agrees with the results shown in Table 3 that pedestrians tend to jaywalk when there is a traffic gap. The phenomenon may indicate insufficient crossing facilities to various destinations on both sides of the streets. Vehicle drivers who frequent such street segments should pay more attention to crossing pedestrians. The second type of jaywalking reflects a mismatch between the design intention and how pedestrians use the streets. For example, in Fig. 8a, we found that in Hong Kong, traffic lights would indicate green simultaneously in both directions for pedestrians to cross. Thus, it is expected that pedestrians would directly walk towards the diagonal. In Fig. 8(b, c), pedestrians could walk anywhere given that there is no approaching vehicle. The last type of observed jaywalking reveals the inconsistency of road signage design. In Hong Kong, the very narrow road sometimes does not have a painted zebra crossing. Occasionally, people read the precautionary pedestrian crossing marks which remind pedestrian to watch left or watch right before crossing. These variations in crossing facilities are legacy inherited from different periods of street network design, which requires further policy interventions to avoid confusion. And a clear policy that traffic must stop for crossing pedestrians in these narrow streets must be conveyed to drivers and enforced to ensure pedestrian safety.

## Conclusion and future research directions

Using street life to reflect a city’s quality is not new in urban studies and transportation planning. However, systematically measuring spatial-temporal pedestrian activities has been challenging. Today, ubiquitous sensors and mobile devices allow researchers to trace pedestrian movement through the veins of the city at a human scale. In this paper, we reviewed recent work using GPS data, video footage, Wi-Fi, and Bluetooth sensors to measure pedestrian activities from the lens of safety, comfort, and convenience. We argue that these data open the door for researchers to reimagine the public realm in a pedestrian-oriented paradigm, uncover the pitfalls in the existing urban environment, and revisit traditional planning theories.

Opportunities coming along with the smart data are three-folded. First of all, the availability of high computation power and advanced algorithms further enhances researchers’ ability to streamline the data collection and analysis process in quantifying fine-grained spatial-temporal patterns. Video-based research, which largely relies on manual tagging, is further empowered by computer vision and deep learning algorithms. Secondly, data vendors, such as Safegraph^9^, Cuebiq^10^ and Facebook’s Data for Good group^11^, have released pre-processed and anonymized data for researchers to use in research directly. This largely reduces the technical barriers of data collection and aggregation. Lastly, smart data provides new collaborative opportunities for academic-industry-government collaboration in testing out urban intervention measures before implementation.

However, our review shows that leveraging these opportunities is not without challenges. First of all, although the development in object detection, instance segmentation, and object tracking algorithms allow researchers to extract pedestrian counts, walking speed, and trajectories from videos, important features such as pedestrian age and gender still require intensive manual labeling. The process of manual labeling is both costly and time-consuming. The process may also introduce bias or errors. Secondly, the analysis of pedestrian activities by advanced computational science is still in its infancy. Algorithms detecting pedestrian interaction and grouping from video, Bluetooth, and Wi-Fi still deserve further improvement. Moreover, there are challenges of relating the environmental components to the type and intensity of pedestrian interaction. Pedestrian perception, including traffic safety, crime safety, and pleasantness, also lacks consistent measures and requires further study.

Additionally, several environmental component variables have been discussed in previous walkability studies. Yet, there is a lack of empirical studies using smart data to support them. For example, pavement and design elements such as effective sidewalk width, availability of accessible ramp, and street furniture are crucial in enhancing a pedestrian-centered street space. These variables are generally hard to measure across a large region.

Another potential challenge lies in using these smart data for transforming the research findings to real-world decision-making. For example, passively collected data such as GPS data from smartphones and video data are subject to sample bias either in which devices or where the camera is installed. When researchers focus on developing projects based on finer spatial and temporal units, these may be inherent demographic biases.

## Data Availability

The bus videos are proprietary materials of the Kowloon Motor Bus Company (1933) Limited (KMB). The authors do not have the right to share the data.

## Footnotes

1 https://www.metmuseum.org/art/collection/search/437310

2 Keywords include: pedestrian crossing, pedestrian trajectory, route choice, desired path, pedestrian safety, pedestrian perception, street crime, wayfinding, pedestrian navigation, travel distance, physical activity level, pedestrian interactions, pedestrian grouping

3 https://github.com/animikhaich/Real-Time-Face-Anonymizer

4 http://cocodataset.org/

5 According to the ResNet18 prediction model specification, our overall pixel-wise accuracy was approximately 78.64%

6 https://www.openstreetmap.org/

7 https://www.openrice.com/en/hongkong

8 The videos provided are sampled at 10 frame per second

9 https://www.safegraph.com/academics

10 https://www.cuebiq.com

11 https://dataforgood.facebook.com/dfg/covid-19
